# The emphysematous lung is abnormally sensitive to TRAIL-mediated apoptosis

**DOI:** 10.1186/1465-9921-12-105

**Published:** 2011-08-08

**Authors:** Mathieu C Morissette, Julie Parent, Julie Milot

**Affiliations:** 1 Centre de recherche de l'Institut universitaire de cardiologie et de pneumologie de Québec (CRIUCPQ), Québec, Canada

**Keywords:** Apoptosis, COPD, oxidative stress, p53, TRAIL

## Abstract

**Background:**

Alveolar apoptosis is increased in the emphysematous lung. However, mechanisms involved are not fully understood. Recently, we demonstrated that levels of TRAIL receptor 1 and 2, levels of p53, and Bax/Bcl-x_L _ratio were elevated in the lung of subjects with emphysema, despite smoking cessation. Thus, we postulate that due to chronic pulmonary oxidative stress, the emphysematous lung would be abnormally sensitive to TRAIL-mediated apoptosis.

**Methodology:**

A549 cells were exposed to rTRAIL, cigarette smoke extract, and/or H_2_O_2 _prior to caspase-3 activity measurement and annexin V staining assessment. In addition, freshly resected lung samples were obtained from non-emphysematous and emphysematous subjects and exposed *ex vivo *to rTRAIL for up to 18 hours. Lung samples were harvested and levels of active caspase-3 and caspase-8 were measured from tissue lysates.

**Results:**

Both cigarette smoke extract and H_2_O_2 _were able to sensitize A549 cells to TRAIL-mediated apoptosis. Moreover, following exposure to rTRAIL, caspase-3 and -8 were activated in lung explants from emphysematous subjects while being decreased in lung explants from non-emphysematous subjects.

**Significance of the study:**

Alveolar sensitivity to TRAIL-mediated apoptosis is strongly increased in the emphysematous lung due to the presence of oxidative stress. This might be a new mechanism leading to increased alveolar apoptosis and persistent alveolar destruction following smoking cessation.

## Introduction

Emphysema, largely caused by cigarette smoking, is mainly characterized by a loss of alveolar integrity leading to poor gas exchange between the alveolar space and pulmonary capillaries [1]. Moreover, the emphysematous lung is an inflamed tissue in which activated neutrophils, alveolar macrophages and lymphocytes are found in large numbers [2]. In addition to proteases and inflammatory mediators, neutrophils and macrophages generate reactive oxygen species (ROS) [3,4]. This adds to oxidative stress aggression induced by primary cigarette smoke exposure and is responsible for the persistence of oxidative stress after smoking cessation [5].

High oxidative stress can damage cell lipids, proteins, and nucleic acids [6]. If too severe, such damage will force cells to activate their programmed cell death (apoptosis) [7]. Exposure to exogenous ROS acts on several apoptosis/survival-related signaling pathways such as MAPK, AKT, JAK/STAT, NF-κB and the DNA damage checkpoint involving p53 [7].

The transcription factor p53 is an important member of the cellular response to DNA damage. Depending on the severity of the DNA injuries, p53 will transcribe genes that will stop the cell cycle (*i.e. p21, 14-3-3σ*) and allow DNA repair. However, p53 can also promote the transcription of pro-apoptotic genes (*i.e. Bax, PUMA, NOXA, Fas, TRAIL-receptors 1 and 2*) that will activate apoptosis and lead to cell death [8].

It is now well accepted that apoptosis is increased in the emphysematous lung [9-12], however, the cause is not fully understood. Our laboratory has previously demonstrated that a sub-lethal dose of hydrogen peroxide (H_2_O_2_) activates p53 and up-regulates Bax and pro-apoptotic TRAIL-receptors (TRAIL-Rs) 1 and 2 in lung adenocarcinoma cells A549 [13]. These apoptotic factors were significantly increased in the lung of emphysematous subjects compared to normal smokers and non-smokers, despite smoking cessation [13].

As the influence of elevated levels of p53, Bax, and TRAIL-Rs in the emphysematous lung on TRAIL-mediated apoptosis sensitivity is not known, we hypothesize that by up-regulating pro-apoptotic factors and TRAIL-Rs, sub-lethal oxidative stress may sensitize alveolar cells to the death ligand TRAIL.

## Methods

### Cell culture and stimulations

A549 cells (human lung adenocarcinoma derived cell line) were obtained from American Type Culture Collection (ATCC, Manassas, VA) and grown in DMEM with 10% fetal bovine serum (FBS) (Cansera, PAA Laboratories, Etobicoke, ON, Canada). Cells were grown in 12-well culture plates to confluence at 37°C in the presence of 5% CO_2 _and starved overnight in serum-free medium before stimulations. After medium was replaced with fresh serum-free DMEM, cells were exposed to 500 μM H_2_O_2 _or 5% cigarette smoke extract (CSE) (prepared according to Proulx et al. [14]) and/or recombinant TRAIL (rTRAIL) (Millipore, Billerica, Massachusetts) 30 and 100 ng/ml alone or with 5 mM N-acetyl-L-cystein (NAC) (antioxidant) (present 30 min before and during rTRAIL/H_2_O_2_/CSE treatment) in serum-free medium. Cells were harvested 6 h after stimulation for caspase-3 activity assay and after 24 h for Annexin V staining. Each experiment was repeated three times.

### Human lung tissues

Fresh human lung tissues were obtained from subjects undergoing lung resections for tumor with or without lung volume reduction. Lung specimens were located far from the tumor and verified by a pathologist to ensure that no tumor tissue was remaining. We obtained lung tissue specimens from 13 different subjects divided in two groups: subjects without airway obstruction or emphysema (n = 7) and subjects with airway obstruction and emphysema (n = 6). Presence of airway obstruction was based on FEV_1 _value and its reversibility following bronchodilator administration. All subjects were between 50 and 75 years old, with a smoking history greater than 15 packs-years. Subjects were matched for age, sex and smoking history. Presence of emphysema was confirmed by high resolution computed tomography scan (CT scan) analysis for all subjects with emphysema and by a pathologist for lung specimens. The "Institut universitaire de cardiologie et de pneumologie de Québec" (IUCPQ) Research Ethics Committee approved the study and all subjects provided written consent.

### Lung explants culture and stimulation

Lung specimens were maintained in cold serum-free Dulbecco's modified Eagle medium (DMEM) (Invitrogen, Burlington, ON, Canada) following resection. Specimens were carefully cut (only parenchyma; no bronchi or pleural tissue) into approximately 9 mm^3 ^explants, placed in collagen-coated 6-well culture plates (3/well) with serum-free DMEM only to prevent explants from drying and incubated 2 h to allow adherence of explants to collagen. Medium was then removed and new medium with or without 100 ng/ml of human rTRAIL was added to cover the explants (rTRAIL dose was determined after *in vitro *experiment). Explants were cultured for 0, 6, 12 and 18 h before harvesting. For each condition, two explants were used for total protein extraction and one embedded in OCT for cryosectioning.

### Caspase-3 activity assay

Caspase-3 activity was measured in cultured lung explants and A459 cells with the Caspase-3 Fluorometric Assay Kit (BioVision, Mountain View, CA) according to manufacturer's specifications. For lung explants experiments, tissues were homogenized in the provided lysis buffer using glass beads (2 mm diameter) for 1 h before total protein concentration was measured (DC Protein Assay; Biorad, Hercules, CA). 40 μg of total protein was used to measure caspase-3 activity in lung explants lysates. For *in vitro *experiments, cells were lysed in the provided buffer before total protein concentration was measured. 50 μg of total protein was used to measure caspase-3 activity in cell lysate. Each measurement was done in duplicate.

### Flow cytometry analysis of annexin V binding

Annexin V binding analysis was used to identify dead and dying A549 cells through both apoptosis and necrosis. A549 cells were trypsinysed (Trypsin 0.25%, EDTA 2.2 mM) and incubated with Annexin V-FITC according to manufacturer's instructions (BD Biosciences, Mississauga, ON, Canada). At least 5000 cells were analyzed by flow cytometry on a Coulter EPICS XL-MCL flow cytometer (Beckman-Coulter; Mississauga, ON, Canada) with the EXPO 32 APC XL 4 Color program (Beckman-Coulter).

### Western Blot

To determine activation of the extrinsic apoptotic pathway, caspase-8 activation was assessed by Western blot in cultured lung explants. 40 μg of protein was loaded in each lane and electrophoresed through 12% SDS-polyacrylamide gels followed by electrotransfer onto a nitrocellulose membrane. After staining with Ponceau Red to ensure that the same amount of protein was transferred onto the membrane, the membrane was incubated for 1 h in 5% fat-free dry milk powder in TBS and 0.05% Tween-20 (TBS-T) at room temperature (RT). The membrane was then incubated with the rabbit anti-human caspase-8 (1/1000) (BD Biosciences, Mississauga, ON) in 5% fat-free dry milk powder in TBS-T overnight at 4°C. Washes were done in TBS-T for 30 min. The membrane was then incubated with the horseradish peroxidase-conjugated goat anti-rabbit IgG (1/5000) (Cell Signaling Technology, Danvers, MA) diluted in 5% fat-free dry milk powder in TBS-T for 45 min at RT. The membrane was washed for 30 min in TBS-T. Bands were revealed by chemiluminescent substrate addition according to manufacturer's insctructions (PerkinElmer, Woodbridge, Ont, Canada). Blots were then exposed to Bioflex MSI films (InterSciences, Markham, Ont, Canada) with intensifying screen. Bands were quantified by densitometry using Image J software (National Institutes of Health, USA).

### Histological analyses

TUNEL and 4-hydroxy-2-nonenal (HNE) stainings, in addition to alveolar density index (ADI) determination, were performed on 8 μm thick OCT embedded lung explants sections.

#### TUNEL staining

TUNEL staining was performed with DeadEnd Colorimetric TUNEL System (Promega Corp., Madison, WI) with modifications to manufacturer's specifications. Following rTdT treatment, sections were blocked with 2% BSA for 1 h at RT. Tissue autofluorescence was blocked with 0.1% Evan's blue (30 min, RT), and sections were then incubated with Streptavidin conjugated to Alexa 488 (0.01 mg/ml, 30 min, TP; Life Technologies, Carlsbad, CA), counterstained with DAPI (10 μM, 15 min, RT) and analysed by fluorescence microscopy.

#### 4-hydroxy-2-nonenal (HNE) staining

Sections for HNE were fixed with acetone/methanol (60/40) for 10 min at -20°C. Endogenous peroxydase was blocked with 0.3% H_2_O_2 _for 30 min at RT. Staining steps were performed with the Vectastain Elite ABC kit (Vector Laboratories, Burlingame, CA) according to manufacturer's specifications. The primary antibody used was a rabbit anti-HNE (1/10 000) (Calbiochem EMD Chemicals, Gibbstown, NJ) O/N at 4°C.

#### Alveolar density index (ADI)

For each lung specimen, three 40 × pictures were taken from one 8 μm thick OCT cut (hematoxylin & eosin stained). A 20 000 pixels grid (sides of 141.42 pixels) was superimposed over each picture (average of 20 squares/field) using Image J software. The number of alveoli walls crossing horizontal and the vertical lines was then counted and expressed as "intercepted alveolar wall/1000 linear pixels". A lower ADI indicates a more enlarged alveolus.

### Statistical Analysis

Data from A549 cell stimulations were compared using one-way analysis of variance (ANOVA) followed by, if p < 0.05, a post-hoc Tukey-Kramer test. Data from subjects and cultured lung explants were compared using unpaired two-sided T test. Correlations were evaluated using Pearson's test and the significance using a one-sample T test. A significant difference was assumed when p values were lower than 0.05.

## Results

### In vitro study

#### H_2_O_2 _and CSE sensitize A549 cells to TRAIL-mediated apoptosis

**Figure 1:**
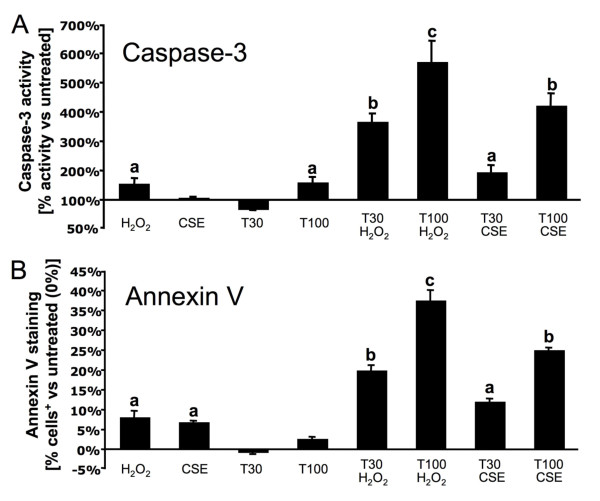
**H_2_O_2 _and CSE sensitize A549 cells to TRAIL-mediated apoptosis**. A549 cells were exposed to 500 μM hydrogen peroxide (H_2_O_2_), 5% cigarette smoke extract (CSE) and/or 30-100 ng/ml of recombinant human TRAIL (T30-T100) for [A] 6 h (caspase-3 activity) or [B] 24 h (Annexin V staining) in serum free media. Experiments were repeated three separate times. Results are expressed as means ± SEM. Bars with different superscripts are significantly different (p < 0.05).

A549 cells are resistant to TRAIL-mediated apoptosis up to 100 ng/ml as shown in Figure 1A and 1B. Moreover, rTRAIL 30 ng/ml reduces baseline active caspase-3 by 30% (Figure 1A). H_2_O_2 _(500 μM) or CSE (5%) treatment alone mildly activated caspase-3, 154% and 106% of control respectively, and did not induce cell death in more than 10% of treated cells (Annexin V positive) (Figure 1B). However, exposure to both rTRAIL (100 ng/ml)+H_2_O_2 _or rTRAIL (100 ng/ml)+CSE had synergistic effects on caspase-3 activation, 570% and 420% of control respectively (Figure 1A), and on cell death induction, +37% and +25% annexin V^+ ^cells respectively (Figure 1B), when compared to untreated cells. Moreover, rTRAIL can be added up to 18 h following H_2_O_2 _treatment and still have synergistic effect with H_2_O_2 _on apoptosis induction (*data not shown*).

#### CSE-induced sensitization of A549 cells to TRAIL-mediated apoptosis is decreased by NAC

**Figure 2:**
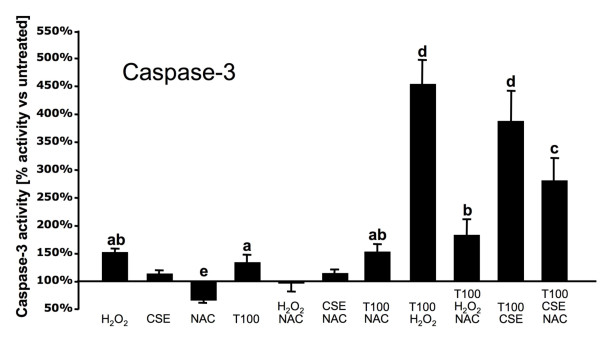
**Oxidative stress is involved in A549 cells sensitization to TRAIL-mediated apoptosis induced by CSE**. A549 cells were pretreated with 5 mM NAC and exposed to 5% cigarette smoke extract (CSE) or 500 mM H_2_O_2 _and/or 100 ng/ml of recombinant human TRAIL (T100) for 6 h in serum free media. Caspase-3 activity was then measured from the whole lysate. Experiments were repeated three separate times. Results are expressed as means ± SEM. Bars with different superscripts are significantly different (p < 0.05).

Exposure of A549 to 5 mM of the ROS scavenger N-acetyl-L-cystein (NAC) 30 minutes before and during exposure to H_2_O_2 _totally abrogated its TRAIL-sensitizing effect on A549 cells (Figure 2). However, NAC treatment only decreased the synergistic effects of CSE and rTRAIL on caspase-3 activation by 27% (Figure 2). Presence of NAC during the exposure (not only pretreatment) to H_2_O_2_/CSE and TRAIL is necessary to limit caspases-3 activation (data not shown).

### Ex vivo study

#### Clinical findings

Characteristics of the subjects and resected lung tissues are presented in Table 1 and Table 2 respectively. The mean age and smoking history of the two groups were similar. Subjects were mostly ex-smokers (one active smoker in each group). Subjects with emphysema had moderate airway obstruction with a mean FEV_1 _at 50% of predicted value. Diffusion capacity of carbon monoxide (DL_CO_) was slightly reduced in subjects with emphysema with a mean value at 77% of predicted. Subjects without emphysema had normal lung function. The presence of emphysema was confirmed by CT scan and by the pathologist for resected lung specimens. Alveolar density index (ADI) was significantly lower in lung specimens from emphysematous subjects than in non-emphysematous ([intercepted alveolar wall/1000 linear pixels] 12.4 ± 2.2 vs 18.7 ± 3.3; p < 0.05) (Table 2). HNE staining score (marker of oxidative stress) was significantly higher in lung specimens from emphysematous subjects than in non-emphysematous (4.2 ± 0.4 vs 2.8 ± 1.3; p < 0.05) (Table 2).

**Table 1:** Subjects' characteristics

Variables	Non-emphysematous subjects *(n = 7)*	Emphysematous subjects *(n = 6)*
Age, yr	72 +/- 6	66 +/- 8
Sex, female/male	4/3	3/3
FEV1, % predicted	97 +/- 17	50 +/- 22*
FEV1/FVC, %	71 +/- 6	45 +/- 11*
DLCO, % predicted	93 +/- 22	77 +/- 36*
Smoking history, pack-year	52 +/- 27	58 +/- 34
Current/ex-smokers	1/6	1/5
Presence of emphysema (CT Scan)	-	6

* Significantly different from non-emphysematous subjects, p < 0.05

Results are presented as mean +/- standard deviation

**Table 2:** Lung tissues' characteristics

Variables	Non-emphysematous tissues *(n = 7)*	Emphysematous tissues *(n = 6)*
Presence of emphysema (determined by the pathologist)	0	6
Alveolar density index (intercepted alveolar wall/1000 linear pixels)	18.7 +/- 3.3	12.4 +/- 2.2*
4-hydroxy-2-nonenal (HNE) staining score (0 = no staining; 5 = max. staining)	2.8 +/- 1.3	4.2 +/- 0.4*

* Significantly different from non-emphysematous tissues, p < 0.05

Results are presented as mean +/- standard deviation

- See Figures 4A and 4B for graphic presentation of Alveolar density index and 4-HNE staining score

#### *Ex vivo *exposure to rTRAIL induces apoptosis in lung explants from emphysematous subjects

**Figure 3:**
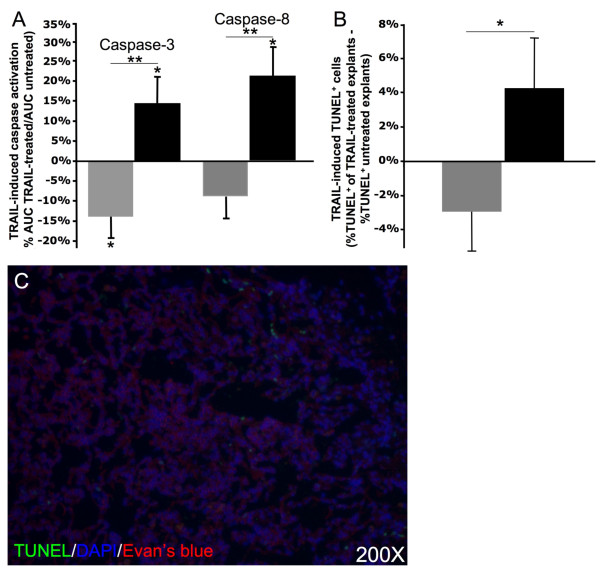
**The emphysematous lung is abnormally sensitive to TRAIL-mediated apoptosis ex vivo**. [A] Caspase-3 activity and active caspase-8 levels were assessed at 0, 6, 12 and 18 h in untreated and rTRAIL-treated lung explants from non-emphysematous (grey) and emphysematous subjects (black). For every subject, values obtained were used to determine the area under the curve (AUC) for untreated and rTRAIL-treated explants. The percentage of variation of the AUC was then determined for every subject ([AUC rTRAIL-treated/AUC untreated]*100). A positive percentage means that rTRAIL induced caspases activation. [B] TUNEL staining was performed on explants from every subject after 18 h of TRAIL treatment or culture control. Every TUNEL^+ ^(apoptotic) and DAPI^+ ^(nucleus) cells were counted and then the percentage of TUNEL^+ ^was determined for every condition ([TUNEL^+^/DAPI^+^]*100). For every subject, the effect of 18 h treatment of TRAIL on apoptosis induction was then determined as follow: %TUNEL^+ ^TRAIL-treated - %TUNEL^+ ^untreated. [C] Representative TUNEL staining of a lung explant used to measure TRAIL-induced apoptosis *ex vivo*. Results are expressed as means ± SEM. *p < 0.05, **p < 0.01.

Activity of caspase-3, a terminal caspase, and protein levels of active caspase-8, a caspase activated by death receptors such as TRAIL-R1/2, were measured to evaluate the effect of rTRAIL on apoptotic pathways activation (Figure 3A). Both caspases were elevated in lung explants with emphysema following 18 h of culture with rTRAIL compared to untreated lung tissues ([Area Under the Curve (AUC) rTRAIL treated/AUC untreated*100] Casp-3 +14.1%; Casp-8 +20.7%). However, caspases induction was not observed in non-emphysematous tissues ([AUC rTRAIL treated/AUC untreated*100] Casp-3 -13.8%; Casp-8 -9.3%) and was markedly decreased (Figure 3A).

**Figure 4:**
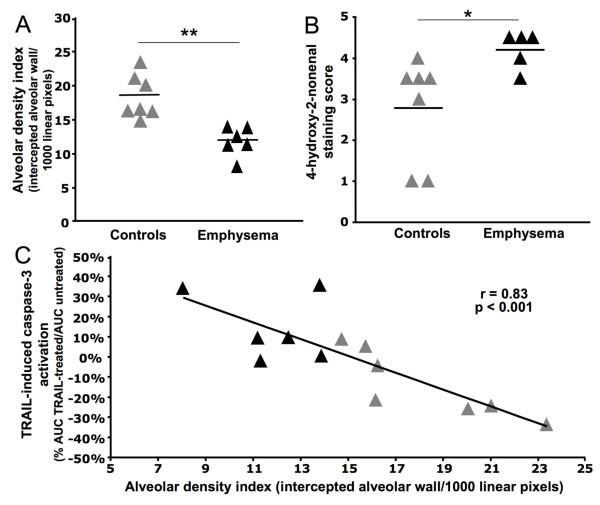
**Sensitivity to TRAIL-mediated apoptosis correlates with lung destruction**. [A] Alveolar density index was measured for every lung sample obtained. Bars represent the means. [B] 4-hydroxy-2-nonenal staining was performed for every subject (except one emphysematous subject) and blindly scored for staining intensity. Bars represent the means. [C] Correlation between TRAIL-induced caspase-3 activation in explants from every subjects and the alveolar density index values. Grey triangles represent non-emphysematous subjects and black triangles represent emphysematous subjects. *p < 0.05, **p < 0.01.

In lung explants cultured for 18 h with rTRAIL, the number of cells undergoing apoptosis (TUNEL^+ ^cells) was increased in explants from emphysematous lung ([%TUNEL^+ ^TRAIL-treated - %TUNEL^+ ^untreated] +4.3 ± 2.9%) and reduced in explants from non-emphysematous lung (-2.9 ± 2.4%) (Figure 3B). Moreover rTRAIL-mediated caspase-3 activation correlated negatively with ADI (r = 0.83, p < 0.001) (Figure 4C), but not with HNE staining (r = 0.20, p = NS).

## Discussion

We previously reported that p53 levels, TRAIL-R1/2 levels and Bax/Bcl-x_L _ratio were higher in the lung of emphysematous subjects as well as in A549 cells exposed to H_2_O_2 _and concluded that it might affect alveolar sensitivity to TRAIL-mediated apoptosis [13]. In line with those results, the present manuscript demonstrate that A549 cells exposed to H_2_O_2 _or CSE are susceptible to TRAIL-mediated apoptosis. This supports our main finding that *ex vivo *exposure of emphysematous lung explants to rTRAIL induced caspases activation and cellular death while rTRAIL had anti-apoptotic properties in non-emphysematous subjects. Moreover, caspase-3 activation following rTRAIL treatment of lung explants correlated with alveolar density index (ADI). To the best of our knowledge, we are the first to report an increased sensitivity of the emphysematous lung to TRAIL-mediated cell death. This work identifies the increased sensitivity to TRAIL-mediated apoptosis as a mechanism for persisting alveolar destruction in the emphysematous lung after smoking cessation. Furthermore, using lung explant culture to test our hypothesis allowed us to study a functional characteristic of the human emphysematous lung.

Our most important finding was that rTRAIL treatment had differential effects on apoptosis induction depending on the presence or absence of emphysema in lung explants. In fact, despite significant smoking history, no apoptotic effect in response to TRAIL was observed in non-emphysematous subjects. Unexpectedly, in addition to rTRAIL having no apoptotic effect, it initiated an anti-apoptotic response. This is likely because TRAIL is able to activate transcription factor NF-κB and AKT, as it has been demonstrated *in vitro *[15]. These signaling pathways are known to promote cell survival directly [16] or indirectly [17] through anti-apoptotic factors such as cFLIP and members of the "inhibitor of apoptosis" (IAP) family (i.e. XIAP, cIAP1-2 and Survivin) that have the ability to prevent caspase-8 and -3 activation. Thus, TRAIL might activate these anti-apoptotic pathways in non-emphysematous lung explants, explaining the decreased caspase-8 and -3 activity following TRAIL treatment. However, the lungs of subjects with emphysema are susceptible to pro-apoptotic activity of TRAIL at a large scale, despite smoking cessation. Our previous demonstration of high levels of TRAIL-R1/2, p53, and elevated Bax/Bcl-x_L _ratio in the emphysematous lung and in A549 cells exposed to H_2_O_2 _[13] might be responsible for the increased sensitivity to TRAIL-mediated apoptosis. In fact, we demonstrated that H_2_O_2 _alone sensitized A549 cells to TRAIL pro-apoptotic effects. Thus, oxidative stress-induced alterations observed in the emphysematous lung are likely to be responsible for shifting TRAIL signaling from anti- to pro-apoptotic pathways.

Importantly, this study documents a strong correlation between TRAIL-induced caspases-3 activation and the alveolar density index (ADI) in lung explants. Alveolar destruction and the enlargement of the alveolar space are the main characteristics of emphysema. Thus, sensitivity to TRAIL is directly related to alveolar destruction, which strengthen its role in emphysema pathophysiology.

In accordance with increased oxidative markers in the emphysematous lung [5] and as oxidative stress increases TRAIL-R1/2 expression, p53 levels and Bax/Bcl-xL ratio (balance toward apoptosis) in A549 cells [13], we report that H_2_O_2 _and cigarette smoke sensitized A549 cells to TRAIL-mediated apoptosis *in vitro*. In fact, the cellular response to injury seems to be extremely important in allowing the TRAIL signaling pathway to induce apoptosis in various cell types (i.e. CRT-MG, DU-145, PC-3, K562 and U397) [18,19]. Thus, cellular response of the emphysematous lung to injury induced by oxidative stress can lead to an increased alveolar sensitivity to TRAIL-mediated apoptosis.

We also show that preventing oxidative stress can prevent sensitization to TRAIL-mediated apoptosis. *In vitro*, H_2_O_2_-mediated sensitization of A549 cells to TRAIL was abrogated by the antioxidant NAC. However, NAC only partly reduced CSE-mediated sensitization to TRAIL. This suggests that some of CSE effects are mediated by oxidative damages, yet the major effects being mediated by factors that could not be scavenged by NAC. Thus, as oxidative stress observed in emphysema is a strong inducer of TRAIL-sensitivity, non-oxidant molecules contained in cigarette smoke can also sensitize cells to TRAIL and might provide a rationale for cigarette smoke-enhanced progression of the disease in emphysematous smokers compared to those who stopped smoking [20].

A limitation of this study was the restricted use of A549 cells in the *in vitro *experiments. A549 cells are widely used in respiratory research [13,21-23] and are a good model to study resistance to TRAIL as they are known to be resistant to TRAIL-induced apoptosis [24,25]. Moreover, they can respond to oxidative stress [13,26] and display functional apoptosis [24,27].

In this manuscript, and in addition to our previous findings [13], we identify oxidative stress-mediated alveolar cell sensitization to TRAIL as a potential causative mechanism in emphysema-related alveolar destruction. Moreover, this study strengthens the role of oxidative stress in the pathogenesis of emphysema and elucidates another of ROS injurious effects.

## Competing interests

Mathieu C Morissette is the recipient of a Ph.D. studentship from the "Fonds de Recherche en Santé du Québec". Julie Parent declares that she has no competing interest. Julie Milot has received unrestricted research grant from "Groupe de Recherche en Santé Respiratoire/NYCOMED" and from "Réseau de Santé Respiratoire (RSR) du Fonds de Recherche en Santé du Québec".

## Authors' contributions

MCM participated in the design of the study, carried out most experiments and drafted the manuscript. JP performed the tissue staining and helped to draft the manuscript. JM conceived the study and helped to draft the manuscript. All authors read and approved the final manuscript.
